# Does napping enhance the effects of Cognitive Bias Modification-Appraisal training? An experimental study

**DOI:** 10.1371/journal.pone.0192837

**Published:** 2018-02-15

**Authors:** Marcella L. Woud, Jan C. Cwik, Simon E. Blackwell, Birgit Kleim, Emily A. Holmes, Dirk Adolph, Hui Zhang, Jürgen Margraf

**Affiliations:** 1 Mental Health Research and Treatment Center, Department of Psychology, Ruhr-Universität Bochum, Bochum, Germany; 2 Clinical Psychology and Psychotherapy, University of Cologne, Cologne, Germany; 3 University Hospital of Psychiatry Zürich, Department of Psychiatry, Psychotherapy and Psychosomatics, Zürich, Switzerland; 4 Department for Clinical Neuroscience, Karolinska Institutet, Huddinge, Stockholm, Sweden; 5 Department of Neuropsychology, Institute of Cognitive Neuroscience, Faculty of Psychology, Ruhr-Universität Bochum, Bochum, Germany; IRCCS Istituto Delle Scienze Neurologiche di Bologna, ITALY

## Abstract

Posttraumatic Stress Disorder (PTSD) is characterised by dysfunctional appraisals of the trauma and its consequences including one’s own symptoms. Experimental studies have shown that Cognitive Bias Modification—Appraisal (CBM-App) training can reduce dysfunctional interpretations and analog trauma symptoms. One important question is how to enhance the effects of CBM-App. Following work suggesting that sleep has beneficial effects on consolidation processes and can thus improve learning, the present study investigated whether a brief period of sleep (i.e., a nap) enhances the effects of CBM-App. All participants watched a stressful movie as an analogue trauma induction. After that, participants received either positive or negative CBM-App training. Within each training, half of the participants then had a 90-minute nap or watched a neutral movie. Results showed that the CBM training induced training-congruent appraisals. Sleep did not enhance this effect. Participants who slept, however, experienced fewer intrusive memories of the analogue trauma, but this effect was independent of the CBM condition. These results provide valuable information about the effects of sleep during a 90-minute nap period on encoding of analogue trauma and emotional learning in the context of appraisal, and highlight the importance of sleep as a focus for continued research.

## Introduction

Posttraumatic Stress Disorder (PTSD) is one of the most frequent psychological disorders following trauma (Diagnostic and Statistical Manual of Mental Disorders-5th edition; DSM 5, [1]). According to the DSM 5, PTSD is characterized by four symptom clusters: (a) Involuntary memories of the trauma, e.g., intrusions or nightmares; (b) Persistent avoidance of stimuli associated with the traumatic event; (c) Negative changes in cognitions and mood related to the trauma; and (d) Alterations in arousal and reactivity that are associated with the trauma. Following assumptions of information-processing theories (e.g. [2], [3], [4]) dysfunctional appraisals of the trauma and its consequences are core factors in the development and maintenance of PTSD. To illustrate, individuals with persistent PTSD appraise the traumatic event and its consequences in a dysfunctional manner, including cognitions such as ‘The event happened because of the way I acted’ or ‘Having these flashbacks must mean I’m going mad’ [2]. As a consequence, experiencing such dysfunctional appraisals is not only in itself highly distressing, but also drives further posttraumatic stress symptoms such as intrusions. Indeed, various studies have shown that dysfunctional appraisals are associated with and predictive of PTSD diagnosis and severity [5], [6]. Furthermore, proof-of-principle studies have provided evidence for a causal role of appraisals using a computerized bias-learning procedure (Cognitive Bias Modification-Appraisal, CBM-App) to train participants to adopt a positive or negative appraisal style towards an analogue stressful event (distressing films) ([7], [8] and for a review on CBM techniques in PTSD, see [9]). Compared to negative appraisal training, training a positive appraisal style induced more functional appraisals and reduced analogue post-traumatic stress symptoms such as intrusions and intrusions distress (for related results in the context of appraisal training, see e.g., [10], [11]). More recently, the effects of reappraisal training in a sample of refugees experiencing PTSD symptoms to varying extents were examined [12]. All participants were exposed to trauma-related pictorial cues and were instructed to either reappraise their emotions or to suppress them. Results showed that refugees experiencing high levels of PTSD symptomatology in the reappraisal group, compared to those who were in the suppression group, experienced fewer intrusions and their intrusions were also less intense.

On the one hand, reappraisal training holds promise for novel (preventive) treatment development. On the other hand, however, the mechanisms of action of such cognitive training paradigms are currently not well understood, and this particularly hinders research aiming to enhance the training’s effects. CBM-based appraisal paradigms, however, might provide a starting point to investigate this in a structured manner. Generally, CBM procedures may best be defined as associative, emotional learning paradigms [13], during which participants are exposed to an experimentally established contingency between a disorder-relevant stimulus and a response, aiming to manipulate participants’ processing style via learning of the presented contingency [14]. One possible way to improve associative learning during CBM is sleep. Sleep is hypothesized to contribute to the consolidation of emotional learning, most likely via off-line processes that stabilize memories. To illustrate, sleep enhances participants’ memory for emotional but not neutral texts ([15] and see also e.g., [16]). Such findings are also crucial for clinically-relevant learning contexts. During exposure to feared stimuli, for example, patients are exposed to new situations and this emotional learning needs to be consolidated. A study testing the potential therapy-enhancing effect of sleep found that spider-phobic participants who napped after exposure to spiders, compared to those who remained awake, reported less subjective fear of spiders and catastrophic spider-related cognitions ([17], and see also [18]).

The present study combines two lines of research. First, the CBM paradigm targeting appraisals of trauma (CBM-App) has potential as a novel treatment add-on in the context of PTSD, operating through establishing new associative networks. Second, sleep may enhance emotional learning and the benefits of psychological therapy procedures. However, additional evidence is needed. Accordingly, the present study had two primary aims: (i) replication of earlier findings showing that appraisals can be trained and that this affects analog trauma symptoms in a training-congruent manner [7], [8], [11]) evaluation of the enhancing effects of sleep on CBM-App in the context of analog trauma.

In the present study, participants watched a stressful movie as an analogue trauma induction [19], followed by either positive or negative CBM-App training. Within each training condition, half of the participants then had a 90-minute nap and the other half watched a neutral movie. Immediate effects of CBM-App on appraisals were tested before and after sleep/wake, and subsequent effects on intrusions over a further one-week period were assessed via a diary. We hypothesised that the CBM-App training would induce training-congruent appraisals (i.e., more functional appraisal after positive compared to negative CBM-App), and that sleep would enhance this effect (e.g., more functional appraisal in the positive CBM-App sleep than positive CBM-App wake group). We also expected training-congruent effects on intrusions at one-week follow-up, (i.e., fewer intrusions after positive compared to negative CBM-App), with sleep being an enhancing factor (e.g., fewer intrusions in the positive CBM-App sleep than positive CBM-App wake group). Finally, we investigated the hypothesised relationship between appraisal and intrusions, and the potential moderating effect of sleep, across the whole sample by examining whether post-training appraisals would predict intrusions, and whether this would be qualified by sleep. Here, we expected more adaptive appraisal at post-training to be associated with fewer intrusions at follow-up, especially among those participants who slept.

## Materials and methods

### Participants

The tested sample included 105 healthy participants (72 female, *M*_*age*_ = 22.9, *SD* = 3.74). Participants’ eligibility was checked via online screening: Participants were required to be fluent in German; not have any vision-or hearing-related problems; be right-handed; not suffer from a blood, injury or injection phobia; not watch horror movies; score < 19 on the Beck Depression Inventory II (BDI-II; [20]), report no suicidal tendencies, and have not had experienced a serious traumatic life event, verified via the Trauma History Checklist (THC; [21]). Further, participant were required to have trait anxiety scores on the State Trait Anxiety Inventory (STAI-T; [22]) in a medium range, i.e., between 30 and 45 to ensure maximum potential to train biases in a positive versus negative direction [23], and to have no sleeping-related problems, reflected by a score of < 6 on the Pittsburgh Sleep Quality Index (PSQI; [24]).

### Films

#### Stressor films

Stressor films included a compilation of distressing film clips, comprising scenes displaying, for example, serious and life-threatening injuries and violence, which have been shown to elicit acute distress and subsequent intrusive memories in similar research [25]. The total duration of the film was 20 minutes. Participants’ engagement with the clips was assessed by means of an 11-point Likert-scale (0 = *no attention*, 10 = *full attention*).

#### Neutral films

Participants in the wake group watched two neutral films. Both films were checked to ensure that they had no content overlap with the stressor films. On day 1, participants watched a 90-minute documentary about the German North sea („Die Nordsee von Oben“; [26]). On day two, they watched a 90-minute documentary about forest song birds („Die Vogelwelt des Waldes“; [27]).

### Sleep recording

EEG was recorded from 6 scalp locations (F3, F4, C3, C4, O1, O2, as well as M1 and M2) in reference to unilateral mastoid (M1) and digitized with 16 bit using a 32 channel Brain Amp DC amplifier (BrainProducts, Germany). Electrodes were attached according to the international 10/20 system by using a standard sleep cap (Easy Cap, Germany). In accordance with standard guidelines (American Association of Sleep Medicine, AASM, [28]), we additionally recorded facial electromyograms from 3 channels, as well as vertical and horizontal eye movements. Sleep parameters and architecture were scored individually by a trained assistant blind to the study’s goal and experimental manipulations. Data processing and sleep staging was performed according to standard criteria of the AASM [28].

### Cognitive Bias Modification-Appraisal (CBM-App)

The training was translated and adapted from that used previously [7], [8]. Participants were presented with a series of ambiguous, reappraisal-related scripts that ended with a word fragment. Participants were instructed to complete the word fragments by typing in the first missing letter. This produced an outcome consistent with a functional or dysfunctional appraisal of the script depending on training condition (positive or negative). Themes from the ‘Self’ subscale of the PTCI [29] were used to develop the scripts given the specific association of these cognitions with PTSD symptomatology [5], [30], e.g., “Trusting oneself to act appropriately in future” was adapted into: ‘*In a crisis*, *I predict my responses will be h-lpf-l / u-el-ss’* (positive CBM-App: ‘*helpful*’, negative CBM-App: ‘*useless*’). To make sure participants processed the scripts thoroughly, a simple yes/no comprehension question was included after just under half of the sentences (for the example above: ‘Do you believe you will be able to respond in a useful way when there is a crisis?’). There were 72 training sentences and 32 comprehension questions, along with 8 emotionally neutral filler sentences giving a total of 80 sentences presented in blocks of 10.

This is an example of a trial sequence: The incomplete script was displayed on the computer screen. Participants were instructed to proceed by pressing the space bar when they had read the script. After the key press, the text disappeared revealing the word fragment. Participants were then instructed to type in the first missing letter of the fragment as quickly as possible. The completed correct word then appeared on screen. Either a comprehension question followed or a new script was presented.

### Encoding Recognition Task (ERT)

The training’s success was assessed via a two-phase Encoding-Recognition Task (ERT; see [7], [8]). During the encoding-phase, 10 novel ambiguous, reappraisal-related scripts were presented. Each script was introduced with a distinctive title and, unlike the former training items, remained ambiguous (i.e., the word fragment did not resolve the script’s ambiguity). After each script, participants were required to imagine themselves vividly in the situation and rate this using a 10 point scale. In the recognition-phase, the 10 encoding-phase titles were presented again, followed by a set of 4 related sentences. Participants rated how close in meaning each sentence was to the original script of that title using a 4-point Likert scale (1 = *not at all similar* to 4 = *very similar*). There were two target sentences, representing a possible positive and negative interpretation of the original script, and two foil sentences, representing a general positive and negative meaning that did not resolve the script’s ambiguity. A bias index was calculated by subtracting the mean ratings for negative targets from those of positive targets, with positive scores indicating a relative bias for endorsing positive over negative interpretations. As participants completed the ERT twice, there were two sets (order counterbalanced). The ERT and CBM-App training were programmed in Inquisit 3.0 [31].

### Posttraumatic Cognitions Inventory (PTCI; [32])

The PTCI is a self-report measure comprising 36 statements reflecting appraisals surrounding traumatic experiences (e.g. ‘I can’t trust that I will do the right thing’). It contains three subscales: negative cognitions about the Self, the World, and Self-Blame. Higher scores reflect a more dysfunctional appraisal style.

### Intrusion assessment

Two measures assessed intrusive memories. First, a 7 day intrusion diary was used. Intrusions were defined as “any memory of the film (or part of the film) that appear apparently spontaneously in your mind. Do not include any memories of the film that you deliberately or consciously bring to mind”. Participants were also instructed about different possible forms of intrusions, i.e., an image, a thought, or a combination, and asked to specify their intrusions accordingly. Participants reported the intrusions’ contents and how distressing each intrusion was (0 = *not all distressing*, 100 = *very distressing*). Furthermore, the intrusion subscale of the Impact of Event Scale-Revised (IES-R; [33]) was employed.

### Mood

Participants’ state mood (i.e., happiness, depression, anger, anxiety, reversed score for happiness) was assessed using an 11-point Likert scale ranging from 0 (*not at all*) to 10 (*extremely*).

### Voluntary memory of stressor film

This assessment included 7 simple questions about aspects of the stressor films. Each question was followed by two answers and participants had to choose one. Correct answers were coded with ‘1’ and incorrect answers with ‘0’ and a sum score (number of correct answers) calculated.

### Procedure

**Fig 1:**
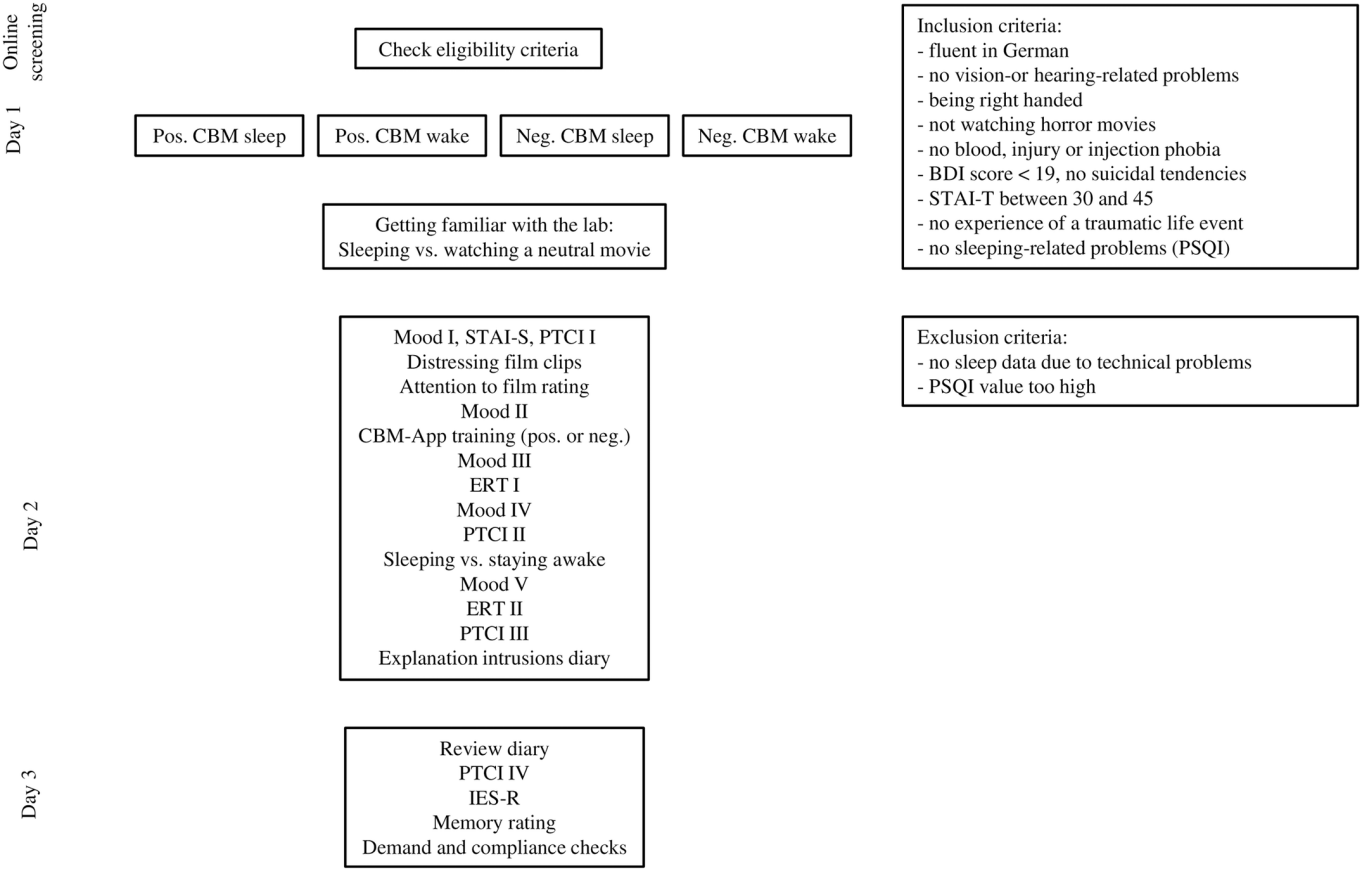
Flowchart procedure. Note: STAI-S: State Trait Anxiety Inventory—State; PTCI: Posttraumatic Cognitions Inventory; ERT: Encoding Recognition Task; IES-R: Impact of Event Scale—Revised.

Participants were screened via an online questionnaire to ensure that they met the inclusion criteria. Eligible participants were then invited to take part in the study. Testing took place between 13:00 and 21:00. Participants were allocated to the four groups via a pre-defined counterbalancing schema. There were three testing days. On day 1, participants signed the informed consent and were made familiar with the lab facilities and the EEG recording. Depending on their experimental condition, participants either slept for 90 minutes or watched a neutral movie while wearing the EEG cap. Participants returned to the lab the following day. This second session started with the baseline measures, i.e., participants’ mood and state anxiety (State Trait Anxiety Inventory—State, STAI-S; [22]) were assessed and participants completed the first Posttraumatic Cognitions Inventory (PTCI; [32]). After that, all participants watched the stressor films. To maximise the impact, participants were left alone in the darkened room to watch the film. Afterwards, participants completed the attention to film rating and the second mood rating. Participants then completed the positive or negative CBM-App training, followed by the third mood rating and the first Encoding Recognition Task (ERT). This was followed by the fourth mood rating and the second PTCI. Participants in the ‘wake’ group then watched a neutral documentary whilst participants in the ‘sleep’ group were instructed to sleep. The time frame for both groups was 90 minutes and participants’ EEG activity was recorded. This was followed by the fifth mood rating, the second ERT, and the third PTCI. The session ended with the explanation of the 7- day intrusion diary. One week later, participants returned to the lab. This third and final session started with reviewing the participants’ diary. Participants then completed the fourth PTCI, the IES-R, the memory rating, and the demand and compliance checks. Finally, participants were debriefed, thanked and paid for their participation (course credit or monetary reimbursement) (see Fig 1 for a diagrammatic overview of the procedure and Supplements for analyses all mood assessments, demands and compliance checks, and full scale and additional subscales IES-R). The study was approved by the ethics committee of the department of psychology at Ruhr-Universität Bochum (approval number: 117), and all participants provided written informed consent to take part in the study.

### Statistical analyses

The sample size was determined via a power calculation based on the effect of positive versus negative CBM on diary intrusions from earlier findings [7]. Intrusion data were reanalysed based on a sample restricted by scores on the STAI-trait, which resulted in a between-group effect size of *d* = .84 [7]. This indicated that *n* = 24 participants per group would be needed to provide 80% power at α = 0.05.

Repeated-measures ANOVAs were conducted to examine changes in appraisal (i.e., ERT and PTCI), with Time x CBM x Group interactions as outcomes of main interest. If significant, t-tests were conducted to further decompose the interaction. Intrusions at follow-up (i.e., diary and IES-R subscale) were compared via CBM x Group univariate ANOVAs. Regression analyses were used to test whether appraisals post CBM training (i.e., ERT II and PTCI II, scores centred) and sleep (coding: -.50 = awake and +.50 = sleep) would predict intrusions assessed at one-week follow-up (i.e., diary and IES-R subscale). Of main interest here was the predictor appraisal and the appraisal x sleep prediction.

## Results

### Participant characteristics

#### Sleep

Prior to analyses, 11 participants were excluded: ten participants had no sleep data because of technical problems and one participant had been tested despite a PSQI score above the cut-off. Accordingly, the final sample was *N* = 94. Following the approach of previous work [17] we intended to include participants of the sleep group if they had reached at least 5 minutes of N2 sleep. However, EEG analyses revealed that only 29 participants had reached this criterion. Hence, in order to preserve sufficient numbers of participants to retain a suitable level of statistical power, we included all participants who reached N1 sleep for at least 5 minutes. In the sleep group, 45 participants reached this criterion, however, two did not. Here, we also followed the approach of earlier work [17] and included participants allocated to the sleep in the wake group for analysis, and vice versa, in case the EEG data did not correspond with a participant’s group allocation (i.e., we were interested in the effect of sleep itself, rather than the effect of allocation to the sleep condition). As such, two participants originally allocated to the sleep group were included in the wake group for analysis. In the wake group, 41 participants stayed awake and 6 participants fell asleep. Hence, these latter 6 participants were thus transferred to the sleep group. Hence, the sample size of the four groups was as follows: positive CBM, sleep: *n* = 24; positive CBM, wake: *n* = 22; negative CBM, sleep: *n* = 27; negative CBM, wake: *n* = 21 (for means, standard deviations and statistic of total sleep duration, see Table 1).

**Table 1:** Baseline and main outcome measures.

		Positive CBM		Negative CBM		Statistics
		Sleep n = 24	Wake n = 22	Sleep n = 27	Wake n = 21	
		20 females 4 males	18 females 4 males	21 females 6 males	13 females 8 males	χ^2^(3): 3.49, *p* = .322
		M (SD)	M (SD)	M (SD)	M (SD)	Main effect CBM
						Main effect Group
						Interaction CBM x Group
Baseline						
	Age	23.50 (3.58)	22.27 (3.36)	23.04 (3.43)	23.52 (4.32)	CBM: *F*_1, 90_ = .27, *p* = .605
						Group: *F*_1, 90_ = .24, *p* = .627
						CBM x Group: *F*_1, 90_ = 1.27, *p* = .262
	STAI-T	37.71 (4.53)	34.68 (3.40)	35.07 (3.58)	35.14 (4.02)	CBM: *F*_1,90_ = 1.80, *p* = .183
						Group: *F*_1,90_ = 3.34, *p* = .071
						CBM x Group: *F*_1,90_ = 3.66, *p* = .059
	STAI-S	34.17 (5.81)	34.41 (6.23)	35.56 (5.64)	34.90 (5.70)	CBM: *F*_1,90_ = .61, *p* = .438
						Group: *F*_1,90_ = .03, *p* = .866
						CBM x Group: *F*_1,90_ = .14, *p* = .713
	BDI	4.29 (3.91)	4.45 (3.70)	4.15 (3.37)	3.57 (4.73)	CBM: *F*_1,90_ = .40, *p* = .529
						Group: *F*_1,90_ = .07, *p* = .799
						CBM x Group: *F*_1,90_ = .21, *p* = .650
	THC	.92 (1.06)	1.23 (1.02)	.93 (1.04)	.76 (1.09)	CBM: *F*_1,90_ = 1.10, *p* = .298
						Group: *F*_1,90_ = .11, *p* = .737
						CBM x Group: *F*_1,90_ = 1.19, *p* = .279
	Distress	12.42 (17.39)	13.68 (14.38)	10.59 (13.82)	15.39 (23.04)	CBM: *F*_1,53_ = .00, *p* = .990
	now*					Group: *F*_1,53_ = .45, *p* = .507
						CBM x Group: *F*_1,53_ = .15, *p* = .698
	Distress	44.82 (31.55)	50.01 (32.69)	35.73 (30.74)	55.53 (33.57)	CBM: *F*_1,53_ = .04, *p* = .836
	then*					Group: *F*_1,53_ = 2.12, *p* = .151
						CBM x Group: *F*_1,53_ = .72, *p* = .399
	Mood 1	12.04 (9.44)	13.64 (8.26)	14.44 (8.70)	15.67 (8.55)	CBM: *F*_1,90_ = 1.49, *p* = .226
						Group: *F*_1,90_ = .60, *p* = .440
						CBM x Group: *F*_1,90_ = .01, *p* = .919
	PTCI I	81.54 (29.19)	92.45 (33.11)	87.04 (25.51)	94.48 (37.53)	CBM: *F*_1,90_ = .34, *p* = .563
						Group: *F*_1,90_ = 2.01, *p* = .160
						CBM x Group: *F*_1,90_ = .07, *p* = .789
	PSQI	3.63 (1.50)	3.73 (.83)	3.63 (1.45)	3.67 (1.28)	CBM: *F*_1,90_ = .01, *p* = .918
						Group: *F*_1,90_ = .07, *p* = .797
						CBM x Group: *F*_1,90_ = .02, *p* = .904
Sleep						
	Total sleep	47.65 (24.00)	.30 (.98)	35.19 (23.44)	.33 (1.06)	CBM: *F*_1,90_ = 2.93, *p* = .090
						Group: *F*_1,90_ = 128.43, *p* < .001
						CBM x Group: *F*_1,90_ = 2.97, *p* = .088
	Sleep stage	23.15 (12.28)	.30 (.98)	19.52 (12.71)	.33 (1.06)	CBM: *F*_1,90_ = .87, *p* = .352
	N1					Group: *F*_1,90_ = 119.93, *p* < .001
						CBM x Group: *F*_1,90_ = .91, *p* = .342
	Sleep stage	12.40 (13.34)	.00 (.00)	9.09 (9.96)	.00 (.00)	CBM: *F*_1,90_ = .86, *p* = .357
	N2					Group: *F*_1,90_ = 36.26, *p* < .001
						CBM x Group: *F*_1,90_ = .86, *p* = .357
	Sleep stage	11.21 (15.23)	.00 (.00)	6.46 (10.26)	.00 (.00)	CBM: *F*_1,90_ = 1.46, *p* = .230
	N3					Group: *F*_1,90_ = 20.28, *p* < .001
						CBM x Group: *F*_1,90_ = 1.46, *p* = .230
	REM sleep	.90 (2.43)	.00 (.00)	.11 (.42)	.00 (.00)	CBM: *F*_1,90_ = 2.30, *p* = .133
						Group: *F*_1,90_ = 3.79, *p* = .055
						CBM x Group: *F*_1,90_ = 2.30, *p* = .133
	Spindel	43.46 (40.79)	.00 (.00)	40.74 (46.11)	.00 (.00)	CBM: *F*_1,91_ = .04, *p* = .839
						Group: *F*_1,91_ = 39.70, *p* < .001
						CBM x Group: *F*_1,91_ = .04, *p* = .839
Attention		9.25 (.79)	9.14 (.89)	9.11 (.93)	8.76 (1.48)	CBM: *F*_1,90_ = 1.42, *p* = .237
to film						Group: *F*_1,90_ = 1.15, *p* = .286
						CBM x Group: *F*_1,90_ = .30, *p* = .586
Memory		5.50 (1.18)	6.00 (1.27)	5.41 (1.12)	5.29 (1.23)	CBM: *F*_1,90_ = 2.65, *p* = .107
stressor						Group: *F*_1,90_ = .58, *p* = .447
films						CBM x Group: *F*_1,90_ = 1.57, *p* = .213
Mood II**		35.88 (21.63)	36.23 (17.28)	32.93 (16.25)	38.52 (19.06)	

Note:

* n deviates depending on whether or not a participant experienced a trauma.

** Because the mood data was analysed pre post the stressor films, i.e., Mood I versus Mood II, we only present the means and standard deviations of the second mood rating. STAI-S / STAI-T: State Trait Anxiety Inventory—State Trait; BDI: Beck Depression Inventory II; THC: Trauma History Checklist; Distress now / then: experienced distress now and then for traumas included in THC; PTCI: Posttraumatic Cognition Inventory; PSQI: Pittsburgh Sleep Quality Index.

#### Baseline measures, compliance and mood change after stressor film

To rule out group differences prior to the CBM training (i.e., gender, age, STAI-T, STAI-S, BDI, PTCI, Mood, THC, PSQI), univariate ANOVAS including the between-subjects factors CBM (positive vs. negative) and Group (sleep vs. wake) were conducted. Results showed no significant interactions or main effects (all *p’s* > .05, except for STAI-T: Group: *p* = .071, Group x CBM: *p* = .059). There were no differences in attention paid to the film and memory ratings of the stressor film (all *p’s* > .05). Finally, a Time (pre film, post film) x CBM (positive vs. negative) x Group (sleep vs. wake) repeated-measures ANOVA showed that participants’ mood had worsened equally after the stressor film, main effect Time: *p* < .001 (see Table 1 for means, standard deviations, and statistics).

### Modification of appraisal: ERT and PTCI

To examine changes in appraisal on the Encoding-Recognition Task (ERT), a repeated-measures ANOVA was conducted including the between-subjects factors CBM (positive vs. negative), Group (sleep vs. wake) and Scenario Set (AB, BA) and the within-subjects factor Time (pre sleep/wake vs. post sleep/wake). Of main interest was the CBM x Group x Time interaction. However, this effect was non-significant, *F*_1,86_ = .01, *p* = .920). Instead, we found a significant CBM x Time interaction (*F*_1,86_ = .9.81, *p* = .002, η^2^ = .102). Two independent t-tests, i.e., one per time point, showed that the CBM training induced training-congruent appraisal styles which differed significantly between the CBM conditions: pre sleep/awake: *t*(92) = 10.93, *p* < .001, *d* = 2.29 (positive CBM: *M* = 1.62, *SD* = .77; negative CBM: *M* = -.88, *SD* = 1.35); post sleep/awake: *t*(92) = 8.13, *p* < .001, *d* = 1.69 (positive CBM: *M* = 1.68, *SD* = .84; negative CBM: *M* = -.03, *SD* = 1.16). Overall, however, sleep did thus not affect participants’ appraisal.

Scores on the Posttraumatic Cognitions Inventory (PTCI) were analysed using two repeated-measures ANOVAs. Both included the between-subjects factors CBM (positive vs. negative) and Group (sleep vs. wake) and the within-subjects factor Time. The first ANOVA compared baseline versus post-CBM PTCI scores (i.e., PTCI I vs. PTCI II). The expected CBM x Time interaction, however, did not reach significance, *F*_1,90_ = 2.95, *p* = .090 (main effect CBM: *F*_1,90_ = 3.36, *p* = .070; main effect Group: *F*_1,90_ = 3.84, *p* = .053). The second ANOVA compared post-CBM PTCI with post-sleep PTCI scores (i.e., PTCI II vs. PTCI III). Here, we expected a significant CBM x Group x Time interaction (i.e., sleep enhancing the difference between the CBM conditions). However, this interaction did not reach significance, *F*_1,90_ = .98, *p* = .326. Instead, there was a significant main effect of CBM, *F*_1,90_ = 5.78, *p* = .018, eta^2^ = .060, showing that in the post-training period (i.e. averaged across PTCI II and PTCI III), participants trained positively had lower PTCI scores than those trained negatively (positive CBM: *M* = 81.73, *SD* = 28.48; negative CBM: *M* = 96.28, *SD* = 30.52). Furthermore, there was a main effect of group, *F*_1,90_ = 4.22, *p* = .043, eta^2^ = .045, with those who slept showing lower scores compared to those who were awake (sleep: *M* = 83.85, *SD* = 29.72; awake: *M* = 95.47, *SD* = 30.05). Overall, there was therefore no differential change in PTCI cognitions between the two CBM training conditions from pre to post training, and sleep did not appear to have any differential impact across the conditions (see Table 2 for all means and standard deviations).

**Table 2:** Changes in appraisal and intrusions at one-week follow-up.

		Positive CBM		Negative CBM	
		Sleep n = 24	Wake n = 22	Sleep n = 27	Wake n = 21
		M (SD)	M (SD)	M (SD)	M (SD)
Appraisals					
	ERT bias index I	1.74 (.72)	1.49 (.83)	-.71 (1.43)	-1.09 (1.27)
	ERT bias index II	1.64 (1.03)	1.71 (.60)	-.01 (1.15)	-.06 (1.20)
	PTCI I	81.54 (29.19)	92.45 (33.11)	87.04 (25.51)	94.48 (37.53)
	PTCI II	78.46 (26.03)	95.36 (32.42)	99.37 (36.37)	107.36 (36.93)
	PTCI III	66.29 (20.09)	88.55 (31.91)	88.74 (31.49)	90.71 (27.14)
	PTCI IV	62.5 (18.04)	85.45 (31.09)	74.96 (25.84)	80.29 (31.05)
Follow-up					
	Intrusions diary*	4.21 (4.30)	5.36 (5.65)	3.96 (3.55)	7.95 (7.60)
	Intrusion distress diary*	34.57 (21.15)	38.12 (23.08)	34.81 (11.92)	39.50 (17.45)
	IES-R intrusion scale	7.58 (5.96)	11.14 (7.21)	7.96 (5.67)	11.57 (5.72)

Note:

* n deviates depending on whether or not a participant experienced intrusions. ERT bias index I: Encoding Recognition Task pre sleep/wake; ERT bias index II: Encoding Recognition Task post sleep/wake PTCI: Posttraumatic Cognitions Inventory, PTCI I: Baseline, PTCI II: post CBM-App, PTCI III: post sleep/wake, PTCI IV: one week follow-up.

### Intrusions: Diary and IES-R intrusions subscale

In total, 82 participants reported intrusions in the diary. We conducted a univariate ANOVA including the between-subjects factors CBM (positive vs. negative) and Group (sleep vs. wake) and expected a CBM x Group interaction. However, this effect was not significant, *F*_1, 90_ = 1.63, *p* = .205. We did find a main effect of Group, *F*_1, 90_ = 5.38, *p* = .023, eta^2^ = .056, showing that overall, those who slept reported fewer intrusions than those who remained awake (sleep: *M* = 4.08, *SD* = 3.88, wake: *M* = 6.63, *SD* = 6.72). Hence, sleep, compared to being awake, led to fewer intrusions. However, this effect was independent of the type of CBM training. Intrusion distress was analysed with the same ANOVA. However, we did not find the expected CBM x Group interaction, *F*_1, 77_ = .02, *p* = .890. The univariate ANOVA examining the IES-R intrusion subscale revealed that those who slept reported fewer intrusive symptoms than those who were awake, *F*_1, 90_ = 7.93, *p* = .006, eta^2^ = .081 (sleep: *M* = 7.78, *SD* = 5.74, wake: *M* = 11.18, *SD* = 6.47), replicating the diary findings. However, the CBM x Group interaction was not significant, *F*_1,90_ = .00, *p* = .983 (see Table 2 for all means and standard deviations).

### Regression analyses to predict intrusions

The first two regressions used diary intrusions as outcome, and Group (sleep vs. wake) and ERT and PTCI appraisals and their interaction as predictors, respectively. The regression including ERT appraisals did not reveal a significant ERT x Group interaction, β = .076, *p* = .462. However, sleep was predictive, β = -.228, *p* = .028. Regarding the regression including PTCI appraisals, we excluded one influential data point (Cook’s D > 1). Results of this regression revealed a non-significant PTCI x Group interaction, β = -.066, *p* = .530, and sleep was also not predictive, β = -.199, *p* = .060. The regression including scores on the IES-R intrusion subscale as outcome revealed the following: If ERT appraisals were entered, there was no significant ERT x Group interaction, β = -.071, *p* = .484. However, sleep was a significant predictor, β = -.284, *p* = .006. If PTCI appraisal were entered, the PTCI x Group interaction was not significant, β = .008, *p* = .936. However, both sleep and PTCI scores were significant predictors, β = -.244, *p* = .016, β = .230, *p* = .024. These data confirm earlier results, i.e., sleep was associated with fewer intrusions whereas appraisals were not, except for PTCI appraisals when predicting the IES-R intrusion subscale. However, given that of the four regressions conducted only one suggested a relationship between appraisals and intrusions, this prediction of IES-R by PTCI should be interpreted with caution.

## Discussion

The current study investigated whether sleep during a 90-minute nap period would enhance learning during an emotional, associative learning task (CBM-App) in analogue traumatic stress. We expected training-congruent changes in appraisal post-training, and that napping would enhance this effect. We expected a similar pattern on intrusive memories, i.e., training-congruent effects on intrusions, enhanced by sleep. Results showed that positive CBM-App, compared to negative CBM-App, led to more functional appraisals. Appraisals measured by the Posttraumatic Cognitions Inventory (PTCI) were more positive across the post-training phase amongst participants who had completed positive CBM-App, compared to those who had completed negative CBM-App. However, sleep did not enhance the training’s effects on appraisals (ERT and PTCI) in either CBM-App condition. Further, there was no difference between the CBM-App conditions on intrusions over the subsequent week. Rather, the nap appeared to have a protective effect regardless of the CBM-App condition: Participants who slept reported fewer intrusions and scored lower on the intrusion subscale of the IES-R compared to those who remained awake. Regression analyses found that whether someone had slept or not to be a stronger predictor of subsequent intrusions than post-training appraisals, and where post-training appraisal did predict subsequent intrusions (in only one of the four regressions conducted), this was independent of the protective effect of sleep during a 90-minute nap period.

Overall, our results partially replicate earlier findings [7], [8] by showing that appraisals of analog trauma can be trained via CBM-App, although unlike these previous studies no differential effect of positive/negative training on downstream analog trauma symptoms (i.e. intrusions or associated distress) were found. Strictly speaking, these results do not provide support for trauma-relevant appraisals playing a causal role in development of trauma symptoms, unlike previous studies found (e.g., [7], [8], [10]). However, these previous studies used designs that were procedurally ‘purer’, i.e., they were operationalized to only test causality without any additional manipulations. Following this, it is possible that the sleep manipulation of the present study added extra variability, making it a less optimal design to find straightforward training effects on analogue trauma symptoms. Further, the protective effect of sleep during a 90-minute nap period may have ‘washed out’ potential effects of CBM over the whole sample. Regarding the results of our sleep manipulation, sleep did not appear to enhance the emotional learning from the CBM paradigm or modulate any subsequent effects of training. There are several explanation that could account for this finding. First, although the sleep group slept for about 40 minutes, not enough participants reached the required 5 minutes of N2 sleep ([17]). Hence, participants were included from 5 minutes of N1 sleep onwards. Both N1 and N2 sleep are considered as early sleep phases (e.g., [34]). However, N2 sleep is the starting phase of the more deeper sleep stages, and consolidation processes have been suggested to occur even in this early stage, e.g., when applying simple motor learning tasks (e.g., [35], [36]). Further, sleep spindles have been observed in N2 sleep which are supposed to play an important role during consolidation, i.e., they support plastic changes in cortical networks (e.g., [34], [37]). Hence, it thus may be that a different pattern of results for our emotional learning paradigm would have been found if higher percentages of N2 sleep would have been reached. This is further supported by correlational data of previous findings showing that higher percentages of N2 sleep were correlated with greater reductions in symptoms from pre- to post-treatment ([17]). Follow-up studies should therefore be designed in such a way that N2 and the deeper sleep phases are more likely to occur, e.g., by testing the effects of a longer nap period or overnight sleep. However, which sleep duration or sleep phases are required to facilitate learning is still a matter of debate. To illustrate, when comparing the effects of a midday nap, hypnosis-relaxation, or wakefulness on a declarative and procedural memory task, results showed that declarative memory was better following nap or hypnosis-relaxation, compared to wakefulness, whereas the performance on the procedural memory task did not differ between the groups [38]. Those who napped slept for about 26 minutes and only reached the early sleep stages (i.e., N1 and N2). Nevertheless, improvements in learning were observed. Most importantly, however, better learning also occurred in the hypnosis-relaxation group in which participants were in fact awake. Hence, although sleep can enhance learning, the exact parameters and mechanisms by which such enhancements may occur are still not completely understood, and will also vary according to learning task used. A second explanation why we did not find an effect on sleep on CBM learning might be a ceiling effect in the ERT data. That is, results on the ERT post CBM / pre sleep showed that a positive bias was induced in those trained positively, and a negative bias in those trained negatively. Hence, it may be possible that there was not much scope for sleep to further enhance the training’s effectiveness. A third explanation could be that the Encoding Recognition Task (ERT) may not have been sensitive enough to capture any effects sleep may have had on learning because of the pre-defined answering format, i.e., rating target sentences. Further, it only included 10 scenarios which also limited the types of cognitions that could be included. Here, follow-up research should test alternative approaches, e.g., approaches that leave more scope for idiosyncratic appraisals and the inclusion of more trauma-related cognitions, such as open-ended scenarios participants have to complete (e.g., [39], [40]). Further, follow-up research could test the effects of sleep on other types of interpretation-based training procedures, e.g., Cognitive Bias Modification—Interpretation (CBM-I) in the context of social anxiety or high trait anxiety (cf. [41]). This would allow testing of whether the present (limited) results are specific for learning during appraisal training or account in general for this type of learning.

However, when looking at the effect of sleep only, i.e., independent of the CBM training, there was a protective effect of the nap in reducing subsequent analog trauma symptoms from the film. These findings replicate those of others who found that napping, compared to being awake, after having watched a stressful movie reduced intrusions [42]. Thus, our findings confirm the idea that following an analog stressor, a brief nap can help reduce the subsequent impact. Theoretically, this may occur because sleep may weaken the negative emotions connected to the films and helps processing and integrating these memories. Interestingly, however, another study found that overnight sleep deprivation (i.e., sleep deprivation for several hours rather than a nap period) resulted in fewer intrusive memories of a stressful film compared to overnight sleep as usual, suggesting that there may be multiple ways in which different aspects of sleep—or lack of it—can have an impact on stressful memories [43]. Hence, follow-up work needs to delineate the precise mechanisms involved, and in particular the timing and amount of sleep (e.g., nap versus several hours; it is only in the latter that consolidation mechanisms would be predicted to occur) that may be beneficial or even harmful. Follow-up work is also needed to further advance our understanding of the theoretical contribution of the effects of sleep on both associative learning and analogue traumatic stress. Since participants did not reach the sleep stage considered to be relevant to consolidate learning during CBM-App, our results are most likely from the direct effect of sleep (i.e., via weakening of the negative emotions connected to the films which enhances their processing) and not per se from the combination of learning and sleep. However, this also makes it very difficult to fully evaluate the role of sleep in the present study. Hence, systematic follow-up work is needed testing different combinations of the trauma film paradigm, CBM-App, and sleep amounts in order to test their individual and combined effects.

To the best of our knowledge, this is the first study testing the effects of sleep on CBM-App. However, in the context of Cognitive Bias Modification—Attention (CBM-A) training, two studies tested the temporal dynamics of learning during CBM-A, and also included a sleep manipulation [44], [45]. In the first study [44], healthy participants were either allocated to an attend-threat training or a control condition. There were two test sessions and three rest intervals: rest condition: session two followed 24h after session one; 1h condition: session two started 1h after session one; no-rest condition: session two immediately followed session one. Results showed that between-session learning only occurred in the two rest conditions, however, this was true for both rest intervals, i.e., 1h and 24h rest. Hence, these data do not support a special role of sleep for learning during CBM but rather emphasize that a brief rest is sufficient to initiate consolidation processes. A second study tested the effects of CBM-A in high and low anxious participants [45]. It included a training to attend either toward or away from threat, and a control training. There were two rest manipulations, i.e., a 24h rest condition and a no-rest condition. Results showed that the performance of highly anxious participants in the avoid-threat condition (i.e., the clinical relevant condition) was comparable to those of the low anxious participants for between- but not within-session learning. Unlike the previous study, these data thus support the role of sleep, showing that even if initial learning is difficult (i.e., when highly anxious individuals train to avoid threat), the learned material can be consolidated via sleep. To conclude, in contrast to our study, both these studies found an enhancing effect of sleep on learning during CBM. At this early stage of research it is difficult to fully understand these findings and several factors could explain them, e.g., the difference in CBM paradigms (attention vs. appraisal training) or sleep manipulation (overnight sleep vs. nap). Hence, follow-up studies are needed that further explore the role of sleep on learning during CBM training.

The present study is not without limitations. A first limitation is the repeated administration of the PTCI. During the second testing day, it was administered three times, and this could have affected the measurement’s accuracy (e.g., via fatigue or carry-over effects). A second limitation is related to our design. In our study, three crucial elements were on the same day, i.e., stressor films, sleep/being awake, and the CBM training. This design was chosen, rather than e.g. having the film on previous day, because moving from the design from earlier studies [7], [8] by having the film and CBM-App on separate days it would make it difficult to interpret any resulting discrepancies between the study results. Nevertheless, our design includes a confound (see, [42], [43]). Third, our power calculation might have overestimated the potential effects, meaning that our study may have been underpowered.

## Conclusions

Sleep (during a 90-minute nap period post film) did not enhance the effects of our cognitive bias modification procedure designed to train positive or negative appraisals of analog trauma. However, we found a protective effect of sleep on subsequent analog trauma symptoms. These results highlight the importance of investigating sleep of varying durations and its potential role in modifying the impact of stressful events.

## Supporting information

S1 File (SAV)

S2 File (DOCX)
