# Supplementary material for: Does napping enhance the effects of Cognitive Bias Modification-Appraisal training? An experimental study
Source: PLoS One. 2018 Feb 15;13(2):e0192837. doi: 10.1371/journal.pone.0192837 (PMC5813960; doi:10.1371/journal.pone.0192837)
Supplement: S2 File — (DOCX) [file pone.0192837.s002.docx]

SUPPLEMENTS: METHODS AND RESULTS

**1. Methods**

*1.1 Demand and Compliance Ratings*

*1.1.1 CBM-App*. To test potential demand effects, at the end of the study participants were presented the following questions: ‘How much do you predict that filling in positive/negative words during the sentence completion task would increase or decrease memories of the negative experience you recorded in your diary?’ and ‘2. How much do you predict that filling in positive/negative words during the sentence completion task would increase or decrease the distress experienced from memories of the negative experience you recorded in your diary?’. Participants of the positive CBM training condition were first asked about the potential effects of filling in positive words, then about the potential effect of filling in negative words. For the negative CBM training condition, the order was reversed. All questions were answered by means of a Likert rating ranging from -10 (*extreme decrease*) to +10 (*extreme increase*).

*1.1.2 Intrusion diary*. Participants were asked to rate their diary completion in relation to the statement, ‘I have often forgotten (or have been unable) to record my intrusive thoughts or images in the diary’, on a scale ranging from 0 (*not at all true of me*) to 10 (*completely true for me*). Furthermore, they had to indicate how accurate they estimated their diary to be, on a scale ranging from 0 (*not at all accurate*) to 10 *(very accurate*).

*1.2 Impact of Event Scale – Revised*

The Impact of Event Scale-Revised (IES-R; Maercker & Schützwohl, 1998) includes 22 items assessing the frequency of current intrusions, avoidance and hyperarousal phenomena tied to a stress-inducing event. The instructions were adapted to reflect experiences linked to the stressor films. Items are rated on a 5-point Likert scale ranging from 0 (*not at all*) to 4 (*extremely*).

**2. Results**

*2.1 Mood Changes*

To investigate mood changes over time, a Time (pre film, post film, post CBM-App training, post ERT I, post sleep/wake) x CBM (positive vs. negative) x Group (sleep vs. wake) repeated-measures ANOVA was conducted. Results showed a significant main effect of Time, *F*_4,87_ = 62.04, *p* < .001, eta^2^ = .074 . However, all other main effects and interactions were non-significant (all *F’s* < 1.80, all *p’s* > .140).

*2.2 Demand and Compliance Ratings*

Univariate ANOVAS including the between-subjects factors CBM (positive vs. negative) and Group (sleep vs. wake) revealed the following for the demand effect questions of the CBM-App training: effect of completing positive words on intrusion frequency: all *F’s* < 2.8, all *p’s >* .100; effect of completing positive words on intrusion distress: main effect of CBM: *F*_1, 89_ = 10.92, *p* < .01, main effect of Group: *F*_1, 89_ = .32, *p* = .573, CBM x Group: *F*_1, 89_ = 2.92, *p* = .091; effect of completing positive words on intrusion frequency: all *F’s* < .90, all *p’s >* .300; effect of completing positive words on intrusions distress: all *F’s* < 1.3, all *p’s >* .250. The analyses of diary compliance and accuracy ratings was tested with the same ANOVA: diary compliance: all *F’s* < 1.0, all *p’s >* .300; diary accuracy: *F’s* < 1.10, all *p’s >* .700. To conclude, these data generally show that there were no differences between the four groups in demand and compliance effects, except for the effect of completing positive words on intrusion distress. Here, we found a main effect of CBM training. However, as the intrusion frequency differed between Group (i.e., sleep vs. wake) and not between CBM training condition (i.e., positive vs. negative), these results do not indicate an effect of demand on the group differences we found (see Supplements Table 1 for means and standard deviations).

*2.3 Impact of Event Scale – Revised*

Results of the CBM (positive vs. negative) and Group (sleep vs. wake) univariate ANOVA on the IES-R total score did not show a CBM x Group interaction, *F*_1, 90_ = .02, *p* = .941. However, we did find a main effect of Group, *F*_1, 90_ = 6.4, *p* < .02, eta^2^ = .066, showing that those who slept reported lower scores than those who were awake (sleep: *M* = 16.02, *SD* = 11.57, wake: *M* = 22.56, *SD* = 13.03). Regarding the analysis of the IES-R avoidance and hyperarousal subscale, results did not show a significant CBM x Group interaction for either scales: avoidance: *F*_1, 90_ = .01, *p* = .912; hyperarousal: *F*_1, 90_ = .18, *p* = .675.

**References**

Buysse, D. J., Reynolds, C. F., 3rd, Monk, T. H., Berman, S. R., & Kupfer, D. J. (1989). The

Pittsburgh Sleep Quality Index: a new instrument for psychiatric practice and research. *Psychiatry Research*, *28*, 193-213.

Ehlers, A. (1999). Posttraumatische Belastungsstörungen. Göttingen: Hogrefe.

Hautzinger, M., Bailer, M., Worall, H. & Keller, F. (1994). *Beck-Depressions Inventar (BDI)*.

Bern: Huber.

Holmes, E. A., & Steel, C. (2004). Schizotypy as a vulnerability factor for traumatic

intrusions: An analogue investigation. *Journal of Nervous and Mental Disease*, *192*, 28-34.

Laux, L., Glanzmann, P., Schaffner, P., & Spielberger, C. D. (1981). *Das State-Trait-*

*Angstinventar (STAI)*. Theoretische Grundlagen und Handanweisung. Weinheim: Beltz Test GmbH.

Maercker, A., & Schützwohl, M. (1998). Erfassung von psychischen Belastungsfolgen: Die

Impact of Event Skala-revidierte Version (IES-R). *Diagnostica*, *44*, 130-141.

*Supplements Table 1*

Demand effects CBM-App training, compliance and accuracy ratings intrusion diary, and Impact of Event Scale – Revised

|  |  | Positive CBM | | Negative CBM | |
| --- | --- | --- | --- | --- | --- |
|  |  | Sleep | Wake | Sleep | Wake |
|  |  | n = 24 | n = 22 | n = 27 | n = 21 |
|  |  | M (SD) | M (SD) | M (SD) | M (SD) |
| Mood | I | 12.04 (9.44) | 13.64 (8.26) | 14.44 (8.70) | 15.67 (8.55) |
|  | II | 35.88 (21.63) | 36.23 (17.28) | 32.93 (16.25) | 38.52 (19.06) |
|  | III | 16.63 (15.43) | 14.86 (9.40) | 18.59 (12.27) | 25.76 (22.55) |
|  | IV | 14.67 (12.07) | 11.54 (7.17) | 16.30 (11.40) | 19.57 (19.19) |
|  | V | 11.13 (12.82) | 6.77 (4.79) | 10.07 (7.56) | 11.43 (7.67) |
| Demand effects CBM-App |  |  |  |  |  |
|  | Positive words & intrusion frequency | -2.83 (2.48) | -1.64 (3.05) | -1.19 (3.33) | -1.24 (3.06) |
|  | Positive words & intrusion distress | -3.58 (2.54) | -2.86 (2.42) | -.42 (3.78) | -1.86 (3.09) |
|  | Negative words & intrusion frequency | 2.38 (2.28) | 1.41 (2.95) | 1.74 (2.26) | 1.76 (2.61) |
|  | Negative words & intrusion distress | 1.83 (1.76) | 1.59 (2.86) | 1.56 (2.87) | 2.52 (2.69) |
| Diary | Compliance | 2.21 (1.69) | 1.95 (1.91) | 1.88 (1.21) | 2.30 (1.56) |
|  | Accuracy | 8.33 (1.81) | 8.36 (1.56) | 8.38 (1.36) | 8.20 (1.58) |
| IES-R |  |  |  |  |  |
|  | Total score | 16.75 (12.92) | 23.05 (12.71) | 15.37 (10.43) | 22.05 (13.65) |
|  | Avoidance subscale | 5.75 (5.36) | 8.41 (6.86) | 5.44 (6.12) | 7.81 (7.17) |
|  | Hyperarousal subscale | 3.42 (5.09) | 3.5 (3.11) | 1.96 (2.07) | 2.67 (3.41) |

Note: Mood: I: pre film, II: post film, III: post CBM-App training, IV: post ERT I, V: post sleep/wake; Demand CBM-App: effect

completing positive words on intrusion frequency, effect completing positive words on intrusion distress, effect completing positive words

on intrusion frequency, effect completing positive words on intrusions distress; IES-R: Impact of Event Scale – Revised.
